# Editorial: Animal models of Alzheimer's disease and other dementias: past, present, and future

**DOI:** 10.3389/fnagi.2024.1539837

**Published:** 2025-01-06

**Authors:** Ines Moreno-Gonzalez, Jesus Garcia-Martin, Roberta Marongiu

**Affiliations:** ^1^Department of Cell Biology, Genetics and Physiology, IBIMA Plataforma BIONAND, School of Sciences, University of Malaga, Malaga, Spain; ^2^Networking Research Center on Neurodegenerative Diseases (CIBERNED), Madrid, Spain; ^3^Department of Neurology, The University of Texas Health Science Center at Houston, Houston, TX, United States; ^4^Department of Neurological Surgery, Weill Cornell Medicine, Cornell University, New York, NY, United States; ^5^Brain and Mind Research Institute, Weill Cornell Medicine, Cornell University, New York, NY, United States; ^6^Department of Genetic Medicine, Weill Cornell Medicine, Cornell University, New York, NY, United States

**Keywords:** animal models, Alzheimer's disease, dementia, risk factors, Parkinson's disease, induced pluripotent stem cell (iPSC), computational models

Neurodegenerative diseases, particularly Alzheimer's disease and related dementias (AD/ADRD), represent a significant global health challenge. As of 2019, dementia affected over 57 million individuals worldwide, with projections suggesting this number could exceed 150 million by 2050 (GBD 2019 Dementia Forecasting Collaborators, 2022). This growing prevalence underscores the need for effective treatments and a deeper understanding of these diseases. Animal models are essential for elucidating the pathology of ADRD, offering valuable insights into disease mechanisms and enabling the evaluation of therapeutic strategies under controlled conditions. Specifically, transgenic models that overexpress human genes linked to familial AD have been instrumental for replicating hallmark features of AD, including the formation of extracellular amyloid-β plaques and intracellular tau neurofibrillary tangles, closely mirroring human neuropathology (Sanchez-Varo et al., 2022). Advancements in gene-editing techniques, such as knock-in models and seeding approaches (Yokoyama et al., 2022), have further enhanced our ability to investigate specific disease mechanisms and track progression with greater precision.

However, traditional animal models often fail to fully capture the complex neurodegenerative processes and diverse risk factors observed in humans, such as aging, genetic predisposition, and lifestyle influences. These limitations highlight the critical need to refine existing models to better incorporate environmental influences, as well as genetic and non-genetic risk factors. In this context, Ganesan et al. introduced a novel mouse model for sporadic AD that combines a knock-in of the APOE^*^4 genetic risk factor with neuroinflammation induced by low-dose intraperitoneal lipopolysaccharide injections. This model revealed early indicators of an AD phenotype, suggesting that further analysis at later stages could uncover additional features of interest.

The importance of models that better reflect human-specific risk factors is further emphasized by the work of Platholi et al., who present in this Research Topic a new model of perimenopausal cerebral amyloid angiopathy to investigate the role of early ovarian decline in neuroinflammatory processes associated with cerebrovascular dementia. In their study, accelerated ovarian failure was pharmacologically induced in transgenic SWDI mice, which express a human amyloid-β precursor protein with vasculotropic mutations (Davis et al., 2004). Their findings revealed dysfunction in key components of the neurovascular unit within hippocampal regions at early stages of pathology, suggesting that the perimenopausal period may represent a particularly vulnerable window for the development of dementia.

As models evolve to better capture the complex risk factors associated with dementia, human stem cell transplantation models offer a promising alternative to address the longstanding challenge of limited translatability from animal studies to clinical applications. In this Research Topic, Ifediora et al. introduce human induced pluripotent stem cell (iPSC) transplantation models as a significant advancement in AD research. By transplanting iPSC-derived neurons, astrocytes, and microglia into animal brains, these models enable the study of human cell interactions within a living brain environment, offering a closer approximation of human disease conditions than traditional isolated cell cultures. This innovative approach, as described by the authors, holds promise for preclinical studies focused on testing and optimizing therapeutic strategies. However, challenges such as replicating long-term aging processes and ensuring stable cell integration remain in these chimeric transplantation systems. Future models emphasizing prolonged observation periods and enhanced cell integration may better capture the chronic and progressive nature of AD and related dementias.

Recent research increasingly focuses on modifiable factors that contribute to dementia risk (Edwards III et al., 2019). In this Research Topic, Perrotta et al. provide a comprehensive overview of genetic, pharmacological, and surgical mouse models to study cognitive decline associated with hypertension, a well-established yet preventable dementia risk (Livingston et al., 2024). Through these models, the authors delve into the mechanisms linking elevated blood pressure to neurodegeneration. While pathogenic pathways vary among these models, the authors highlight several shared, key processes underlying hypertension-induced brain damage, demonstrating the versatility of animal models presented in this mini review.

Beyond hypertension, sleep disturbances are also gaining recognition as a significant risk factor for dementia (Shi et al., 2018). In this Research Topic, Drew et al. examine 15 different mouse models to investigate the relationship between AD and sleep dysfunction. Despite the complexity of sleeping phenomena, these models effectively replicate a wide range of sleep disturbances observed in AD, including alternations in NREM and REM sleep duration, bout lengths, bout counts, and power spectra. Nevertheless, the authors note inconsistencies in analysis methodologies across studies, emphasizing the need for standardized strategies to address these gaps in future research.

In addition to AD and cerebrovascular dementia, two other prevalent neurodegenerative disorders, Parkinson's disease (PD) and dementia with Lewy bodies (LBD), are characterized by dementia symptoms and the presence of pathological aggregates of α-synuclein within the Lewy bodies and Lewy neurites. Haikal et al. review several animal models used to replicate these pathologies, including those employing genetic manipulation, viral vector overexpression, or fibril seed injections to induce α-synuclein accumulation. They highlight how methodological differences, animal species, and α-synuclein variants may contribute to variability in study outcomes. Promisingly, the development of new models that more closely mimic PD and LBD pathophysiology could significantly improve the translational relevance of research in this area.

Beyond traditional, experimental models, predictive models powered by machine learning algorithms represent an emerging frontier in dementia research. These algorithms allow researchers to analyze the interactions between diverse risk factors and dementia progression, integrating findings from both animal models and large patient cohorts. In this case, Yue et al., leveraged data from the China Longitudinal Aging Study to develop an explainable prediction model for AD and mild cognitive impairment. By integrating large datasets, such predictive models provide a framework to understand and validate findings from animal studies, consolidating varied data into a more comprehensive and accurate view of disease mechanisms.

The rising global impact of neurodegenerative diseases, especially AD, highlights the urgent need to develop improved treatments and deepen our understanding of disease neuropathophysiology. Traditional animal models have been invaluable for studying dementia. New and refined animal models that incorporate genetic and environmental risk factors—along with human stem cell and advanced computational approaches—are bridging the gap between animal and human studies. This integrated approach presents a promising future where advanced tools converge offering new opportunities to develop precise and effective interventions for dementia and address this critical public health challenge.
